# Exploring the Role of Food and Food-Related Compounds in Parkinson’s Disease

**DOI:** 10.3390/foods15030514

**Published:** 2026-02-02

**Authors:** Ilaria Trezzi, Gianluca Rizzo, Francesca Giampieri, Maurizio Battino, Luciana Baroni

**Affiliations:** 1 Scientific Society for Vegetarian Nutrition-SSNV, 30171 Venice, Italy; luciana.baroni@scienzavegetariana.it; 2 Department of Clinical Specialistic and Odontostomatological Sciences, University Polytechnic of Marche, 60131 Ancona, Italy; f.giampieri@univpm.it (F.G.); m.a.battino@univpm.it (M.B.)

**Keywords:** Parkinson’s Disease, diet, food, plant-based diet, neuroprotection, gut–brain axis

## Abstract

Parkinson’s disease (PD) is the second most common neurodegenerative disease, characterized by motor and non-motor symptoms that significantly impact patients’ quality of life. Beyond pharmacological treatments, nutrition plays a crucial role in the prevention and management of the disease. Nutritional interventions represent a pivotal strategy for improving clinical outcomes and quality of life in PD patients, addressing issues such as delayed gastric emptying, constipation, weight loss, malnutrition, and chewing or swallowing difficulties. A plant-based diet is particularly suitable for such patients, due to its high fiber content which can enhance gastrointestinal motility, thereby improving levodopa bioavailability, and potentially ameliorateing PD symptoms. For this reason, alongside neurological support, PD patients should receive nutritional counseling. Moreover, food choices can influence the risk of developing the disease: a high consumption of dairy products has been associated with an increased risk of PD; conversely, many plant foods could elicit neuroprotective effects thanks to beneficial phytochemicals such as flavonoids, especially anthocyanins. Furthermore, a moderate coffee consumption could reduce PD risk and progression. The aim of this review is to explore the impact of dietary factors on the risk and progression of PD, evaluate the therapeutic potential of specific foods and dietary patterns in disease management, and highlight the clinical significance of nutritional interventions, specifically focusing on plant-based diets.

## 1. Introduction

**Figure 1:**
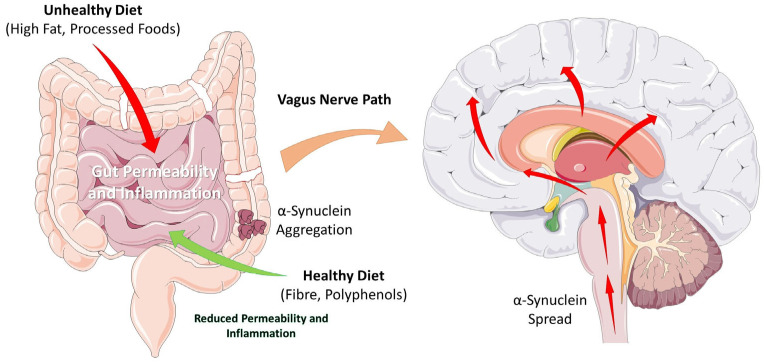
Braak’s Hypothesis: Dietary Impact on Gut–Brain Axis and α-Synuclein. Images provided by Servier Medical Art (https://smart.servier.com, accessed on 21 December 2025), licensed under CC BY 4.0 (https://creativecommons.org/licenses/by/4.0/, accessed on 21 December 2025).

Diet represents a key modifiable environmental factor that influences the onset and progression of neurodegenerative diseases. The Western diet, rich in saturated fats and refined sugars, is associated in humans with impaired memory function [1,2,3], linking this dietary pattern to Alzheimer’s Disease (AD) [4]. Nutrition can also influence the risk of developing Parkinson’s Disease (PD) [5], the second most common neurodegenerative disorder after AD. It affects 2–3% of the population over 65 years of age, with a higher prevalence in North America, South America, and Europe [6]. PD is caused by the degeneration of dopaminergic neurons located in the substantia nigra pars compacta (SNpc) of the mesencephalon. Neuroprotective approaches focus on slowing or arresting the progressive loss of dopaminergic neurons in the SNpc and the associated motor and non-motor symptoms of PD. Neuroprotective strategies aim to reduce oxidative stress, inhibit apoptosis, stabilize mitochondrial function, and prevent α-synuclein aggregation. Most cases are considered sporadic, caused by a combination of genetic and environmental factors that promote the neurodegenerative process. The most important non-modifiable risk factors for developing PD are age, followed by sex and ethnicity [7]. Among modifiable risk factors, diet represents a pivotal one that can contribute to neuroprotection [5]. Food can either be protective or increase the risk of developing PD. α-synuclein is a small protein of unknown biological function that is prone to aggregate into insoluble compounds, resulting in neuronal toxicity. Aggregated α-synuclein in the form of Lewy bodies is the neuropathological hallmark of PD. According to Braak’s theory, α-synuclein aggregates can propagate in a stereotyped manner from the gut to the brain, following a precise scheme [8]. One hypothesis is that α-synuclein fibrils can be transmitted from cell to cell via a “prion-like” spreading mechanism [9]. In support of this hypothesis, evidence shows that a truncal vagotomy (the surgical resection of the vagus nerve) performed on a cohort of Danish patients was associated with a reduced risk of PD [10]. The potential pathway of α-synuclein spread from the enteric nervous system to the brain, modulated by dietary factors, is summarized in Figure 1.

The diagram illustrates how diet can influence disease progression. An unhealthy diet (high in saturated fats and processed foods) can promote gut dysbiosis and increase intestinal permeability (“leaky gut”), triggering neuroinflammation and the misfolding/aggregation of α-synuclein in the enteric nervous system. These aggregates can be retrogradely transported to the central nervous system via the vagus nerve.

Conversely, epidemiological and mechanistic evidence indicates that dietary patterns rich in plant-based foods are associated with a reduced risk of PD onset and slower progression, thanks to a high content of antioxidants, polyphenols, and unsaturated fatty acids with anti-inflammatory properties and beneficial effects on the gut microbiota [11]. Accordingly, it is hypothesized that a healthy, plant-based diet can protect the intestinal barrier, modulate the microbiota, and exert neuroprotective effects, potentially slowing down the neurodegenerative process.

Nevertheless, although most evidence indicates that plant-rich diets might be protective, there is currently no conclusive evidence to recommend a specific dietary pattern for PD prevention; only large, long-term randomized controlled trials can clarify the role of nutrition. Controversies regarding nutrition in PD are mainly due to a focus on individual nutrients, which often lead to inconclusive results, as well as small sample sizes, short durations, and heterogeneity in interventions and outcomes [12]. Moreover, diet can play a fundamental role in the management of PD motor and non-motor symptoms, both at early and advanced stages of the disease. This review aims to evaluate the impact of specific nutritional factors on PD risk modulation and to assess the efficacy of various dietary patterns in optimizing symptom management. In particular, this review will focus on dietary risk factors such as meat, seafood, dairy products, and saturated fat intake, as well as protective factors like flavonoids, coffee, and nicotine in *Solanaceae*. A separate section is dedicated to toxicants, specifically pesticides found in animal-derived foods, while another highlights the importance of maintaining urate levels higher than average to prevent PD.

## 2. Methods

We conducted a comprehensive search on PubMed and Google Scholar databases from inception to 28 October 2025 to identify original studies. The search strategy focused on common risk factors for Parkinson’s Disease, specifically dietary components and lifestyle habits. Key search terms included: ‘Parkinson’, ‘plant-based diet’, ‘vegan diet’, ‘risk factors’, ‘meat’, ‘fish’, ‘seafood’, ‘dairy’, ‘milk’, ‘galactose’, ‘pesticides’, ‘beans’, ‘*Vicia faba*’, ‘*Solanaceae*’, ‘*Mucuna pruriens*’, ‘polyphenols’, ‘antioxidants’, ‘coffee’, ‘tea’, ‘plant foods’, ‘smoking’, ‘tobacco’, ‘nicotine’, ‘fats’, ‘urate’, ‘dietary levodopa’, and ‘dietary dopa’. Inclusion criteria were restricted to original articles in English involving humans or *in vitro* models. Animal studies were excluded, in alignment with recent recommendations regarding human-relevant methodologies in this field [13]. Following duplicate removal, articles were screened by title and abstract; final eligibility was determined after full-text review. Additionally, the reference lists of selected articles were manually screened to identify further relevant studies. A narrative synthesis of the primary findings is presented.

## 3. Meat and Its Derivatives

Some studies have highlighted that frequent consumption of red meat is associated with an increased risk of developing PD [14,15]. Although results are not entirely consistent [16], meat, especially red meat, contains various compounds that might trigger neurodegeneration. Red and processed meats (such as cured meat or corned beef) are rich in saturated fats, which have been linked to an increased risk of PD [17,18,19]. Furthermore, meat processing and cooking may result in toxic compounds such as heterocyclic amines (HCAs) [20]. HCAs have been demonstrated to be mutagenic [21] and to exert neurotoxic effects on *in vitro* dopaminergic neurons [22]. Another interesting hypothesis linking meat consumption to PD is that vertebrates contain α-synuclein in their muscle tissue [23].

However, since many people consume meat while only a small fraction develop PD, it is unlikely that meat consumption represents an independent cause of the disease [24]. Other factors, such as genetic, epigenetic, inflammation, and aging, likely induce dietary α-synuclein to enter the host and initiate the disease. In accordance with the “leaky gut” hypothesis, age-related increases in intestinal permeability facilitate the translocation of bacteria and microbial-derived products; this process is thought to play a pivotal role in the prion-like propagation of α-synuclein aggregates. Furthermore, low fiber intake affects the intestinal barrier and enhances α-synuclein transmigration. PD is often associated with an altered microbiome, which is prone to releasing inflammatory products that can enhance oxidative stress, potentially triggering α-synuclein accumulation in the enteric nervous system (ENS). Bacterial inflammatory products, particularly lipopolysaccharides (LPS), trigger systemic inflammation (mediated by TNF-α, IL-1β and, IL-6) that promotes blood–brain barrier (BBB) disruption and the consequent spread of inflammation to the brain [25]. The gut microbiota of PD patients is often characterized by a depletion of *Prevotella*, known for its fiber-degrading action, and a concomitant enrichment of *Enterobacteriaceae*. Reduced *Prevotella* abundance has been associated with increased gut permeability, which may facilitate α-synuclein aggregation within the enteric nervous system [26]. Long-term fiber consumption is associated with enhanced levels of *Prevotella*, suggesting that a plant-based diet may be beneficial for PD patients. *Prevotella* is not the only genus affected; *Faecalibacterium*, *Blautia*, *Coprococcus*, and *Lachnospiraceae* are typically decreased, while other genera are increased (e.g., *Bifidobacterium*, *Lactobacillus*, *Akkermansia*, *Ruminococcaceae*, *Verrucomicrobiaceae*, and *Christensenellaceae*) [27]. Consequently, PD patients exhibit a distinctive gut microbiome composition compared to healthy individuals. Beyond the neuroprotective properties of phytonutrients, plant-based dietary patterns can induce favorable modulations in the gut microbiota to counteract PD pathogenesis [28]. Several mechanisms are hypothesized to drive the initiation and perpetuation of neurodegeneration in PD. Notably, the gut-to-brain translocation of α-synuclein represents a key pathological pathway, although it requires further empirical validation.

## 4. Seafood and Heavy Metals

Omega-3 polyunsaturated fatty acids (n-3 PUFAs), such as alpha-linolenic acid (ALA), eicosapentaenoic acid (EPA), and docosahexaenoic acid (DHA), are essential components of the central nervous system that maintain neuronal membrane fluidity and signaling [29]. These fatty acids can attenuate neuroinflammation by inhibiting microglial activity and upregulating neuroprotective astrocytic pathways through the secretion of neurotrophic factors [30,31,32]. n-3 PUFAs are primarily found in fatty fish (salmon, sardines, herring, anchovies, tuna) but also in plant foods like flaxseeds, chia seeds, walnuts, and microalgae. Prospective studies using fish oils, which are rich in n-3 PUFAs, have shown that their intake is associated with a decreased risk of developing PD [31,33]. Emerging evidence indicates that the neuroprotective effects of n-3 PUFAs result from combined mechanisms: inhibition of pro-inflammatory cytokines (TNF-α, IL-1β, IL-6) [34], rescue of mitochondrial function, and reduction in oxidative damage [35]. Specifically, DHA (a very-long-chain omega-3 fatty acid) can increase dopamine synthesis by phosphorylating tyrosine hydroxylase [36].

Nevertheless, fatty fish and derived oils are often susceptible to contamination by environmental pollutants, notably heavy metals and persistent organic pollutants. Long-term exposure to certain heavy metals, such as mercury (Hg), cadmium (Cd), and lead (Pb), can induce neurotoxicity even at low concentrations [37]. In particular, Hg and Pb contribute to PD onset and progression, often acting synergistically with manganese (Mn) and copper (Cu) [38]. Metals like Cd, Cu and Hg have a high affinity for the dopamine- 2 receptor [39], specifically binding to free sulfhydryl (-SH) groups with an antagonist effect. Other metals like Mn, Cd and Cu can increase presynaptic α-synuclein aggregation [40]. Heavy metals can also impair blood–brain barrier (BBB) permeability, thereby promoting neurodegeneration [41,42].

In light of these toxicological concerns, sourcing dietary n-3 PUFAs from plant-based alternatives represents a valuable strategy. Plant-based sources include DHA (from microalgae) and ALA (found in flaxseeds, chia seeds, and walnuts) which can be endogenously converted to EPA and DHA [43]. These plant-based sources can provide neuroprotective fatty acids free from environmental contaminants, offering a safer nutritional strategy for PD prevention.

## 5. Dairy Products

The strongest dietary association with an increased risk of PD is the consumption of milk and dairy products [44]. Five large prospective studies have confirmed this association, independent of vitamin D or calcium intake [45,46,47,48]. A meta-analysis of these studies revealed a dose–response relationship between dairy intake and PD [49]. Data from two large prospective studies, including the Harvard Nurses’ Health Study, found a positive association between dairy intake and PD [44]. The exact mechanisms through which dairy products lead to neurodegeneration remain elusive. Some authors attribute this to pesticide contamination [50,51]; evidence suggests that PD motor symptoms typically appear after a critical threshold of 70–80% loss of dopaminergic neurons in the SNpc. A post-mortem study from the Honolulu-Asia Aging Study revealed a positive association between SN neuron loss and milk intake, even after excluding PD and Lewy Body Dementia (LBD) cases. Brains of subjects with the highest milk consumption had higher levels of heptachlor epoxide. It has been suggested that neurodegeneration may stem from potential milk contamination with organochlorine pesticide which are fat-soluble [52]. Indeed, reports of pollutants in milk worldwide highlight the need for regular product screening [50,53,54,55]. However, this hypothesis is challenged by the fact that skimmed milk, which contains less fat and fewer pesticides, has the same effect as other dairy products [56]. Another hypothesis is that dairy may contain traces of α-synuclein, though no confirmation yet exists [57]. Alternatively, high milk consumption provides galactose which, at doses above 100 mg/kg (roughly two glasses of milk perday), can be neurotoxic. SNpc dopaminergic neurons exhibit enhanced vulnerability to oxidative stress, particularly when induced by galactose. Indeed, galactose is a hexose sugar capable of inducing brain aging [58]. Other cohort studies suggest that increased PD risk may be linked to the urate-lowering effects of dairy products [59], as urate is involved in neuroprotection [60]. The exact mechanisms through which dairy products increase PD risk require further investigation.

## 6. Dietary Fat Intake

Dietary fat consumption is hypothesized to contribute to PD pathogenesis through exacerbated oxidative stress, particularly involving high levels of saturated fatty acids. Neuronal membranes are rich in polyunsaturated fatty acids and highly vulnerable to free radicals, which can lead to a peroxidation cascade and neuronal death. It is speculated that saturated fats may alter polyunsaturated fatty acid metabolism and membrane lipid composition [61]. The lipid profile of neuronal membranes is a fundamental factor in determining cellular vulnerability to oxidative damage [62]. Saturated fatty acids are linked to chronic inflammation and oxidative stress [63]. Additionally, pesticides can modulate PD risk via fat intake.

Evidence suggests that individuals with low polyunsaturated or a high saturated fatty acid intake are more vulnerable to striatal neuron damage caused by pesticides, thereby increasing PD risk [18]. Previous findings on the association between dietary fat and PD are inconsistent and fail to demonstrate a clear link for total fatty acids [64,65]. While some studies link PD to total fat, cholesterol, and saturated fats [17], others find a protective effect from high polyunsaturated fatty acid intake [47,65,66]. Despite these inconsistencies [65,67], elevated consumption of saturated fatty acids appears associated with an increased risk of PD onset. In a large 2003 prospective study, this correlation was found only in men, though it was not statistically significant [61]. Other studies have shown similar correlations [18,62]; a recent prospective study on a large Swedish cohort demonstrated that high saturated fat intake was associated with increased PD risk. This association was not confirmed for total, monounsaturated, or polyunsaturated fat intake [19].

In conclusion, while further research is needed, dietary fat composition likely influences the risk of PD. The link between saturated fats, cholesterol, and cardiovascular diseases is well established. It is probable that high saturated fatty acid intake contributes to an increased risk of PD as well. Therefore, reducing saturated fat intake in favor of polyunsaturated fatty acids may benefit overall health and promote longevity through potential neuroprotective effects. Pending definitive evidence, a plant-based dietary pattern offers significant benefits due to the absence of cholesterol, lower saturated fats and higher concentrations of polyunsaturated fatty acids compared to conventional diets.

## 7. Pesticides

One of the most frequent non-motor symptoms in PD is constipation, which may develop years before the appearance of motor symptoms and the diagnosis of PD. A reduction in daily bowel movements is predictive of developing PD [68]. PD patients tend to have a lower water intake, but constipation itself could also lead to PD. It can be speculated that constipation may increase PD risk due to the prolonged retention of feces, leading to greater absorption of neurotoxicants [69]. As a matter of fact, pesticides have been linked to PD, although not all agents used in agriculture have been assessed yet. Most studies focus on occupational exposure compared to dietary exposure. It is well-known that people living in rural areas, who are more exposed to organophosphates, have a higher risk of developing PD [51]. There appears to be a positive association between the duration of pesticide exposure and the risk of developing PD [70]. The main pesticides associated with PD include paraquat, rotenone, and several organochlorines. Organochlorines include dioxins, polychlorinated biphenyls (PCBs), and insecticides such as dichlorodiphenyltrichloroethane (DDT) [71,72]. PCBs are synthetic organic pollutants that were widely used in the past in industrial applications and have now been banned by many countries for health reasons. DDT, a synthetic insecticide previously employed for agricultural applications and the control of vector-borne diseases like malaria and typhus, is now prohibited in numerous jurisdictions due to its environmental persistence and potential toxicity. Although most of these chemicals were banned decades ago, they persist in the environment and contaminate the food chain, accumulating in the fatty tissue of animals and humans. Beyond their established role in carcinogenesis, environmental pesticides have also been implicated in the pathogenesis of PD [73].

While pesticides are toxic because they may cause DNA mutations that increase susceptibility to diseases such as PD [74], they may play a more direct role in destroying dopaminergic neurons by inducing mitochondrial dysfunction, inflammation and epigenetic methylation [75]. A key feature of dopaminergic neurons in the basal ganglia circuit is their reliance on intracellular calcium levels to sustain spontaneous activity: pesticides may interfere with this homeostasis leading to neurodegeneration [75], and may also increase the risk of developing PD through an alteration of the gut microbiome [76].

The majority of pesticides are found in animal products such as fish, meat, and fish oils, but also eggs and dairy, because many of them are fat-soluble; conversely, the lowest levels of contamination are found in plant foods [77,78]. Nowadays, farm animals (originally herbivores) are often fed with slaughterhouse by-products that increase their content of industrial chemicals. For example, tetrahydroisoquinoline is mainly found in cheese, and dioxin also contaminates butter and eggs [79]. Therefore, eating a plant-based diet can confer protection against pesticides and pollutants in general [80].

Unfortunately, many PD-associated toxicant chemicals are environmentally persistent and can also be found in drinking water supplies, especially in rural areas [81]. Beyond occupational exposure where the use of personal protective equipment may reduce the risk of exposure to toxic compounds, a healthy diet rich in plant foods, antioxidants, and phytochemicals, might be of great importance for the general population as a preventive strategy for many diseases, including PD.

## 8. Urate Levels

Uric acid (UA), the end product of purine metabolism in humans, has been associated with PD by many studies. Low blood UA levels have been associated with an increased risk of developing PD, as well as with promoting its progression and severity. This association is strong and highly reproducible. Low UA levels also correlate with some non-motor PD symptoms such as neurocognitive dysfunction, fatigue, and sleep disorders [82,83]. However, the biological mechanisms behind these associations are still elusive. Understanding the nature of this link is fundamental, as it may provide insights into the pathogenesis and eligible treatments for PD. It is supposed that low UA levels predispose individuals to oxidative stress, which may trigger dopaminergic neuron degeneration [84]. According to this theory, treatments that increase UA levels should be protective against PD and slow down disease progression. Unfortunately, a recent clinical trial aimed at raising UA in PD failed to show any benefit [85].

Although the main hypothesis is that low UA levels predispose to oxidative stress and subsequent neurodegeneration, reverse causality cannot be excluded, i.e., that it is PD itself that reduces UA levels. For this reason, numerous prospective studies have been performed and they all demonstrated an inverse relationship between low UA levels and PD; however, a reverse association could not be entirely excluded because the disease begins decades before the appearance of motor symptoms [84]. Mendelian randomization studies have also detected a relationship between low UA levels and PD, but a causal link could not be definitively ascertained. These epidemiological studies could not determine the direction of causation. Many publications have highlighted the antioxidant properties of UA, similar to those of vitamin C [86]. It chelates metals, including iron, which can be toxic to dopaminergic neurons when in excess [87,88]. It was also investigated whether neurons are more exposed to UA, and it was found that UA levels in the brain are much lower than in plasma. Little UA is produced in the brain, and plasma UA does not readily cross the blood–brain barrier (BBB) [89]. Some researchers have analyzed UA levels in post-mortem human brains and found a slight but non-significant reduction in PD patients [90]. Nevertheless, these results must be carefully evaluated, because UA levels degrade rapidly once blood flow to the brain is interrupted [84]. In conclusion, the brain does not produce UA and is not exposed to high UA levels.

Normally, UA levels fluctuate during the day, and consequently, are highly variable in the same individual. They also depend on different factors, including age, sex, body mass, physical activity, food and medications [91]. Diet significantly affects UA levels, as almost all foods contain purines from DNA, RNA, and their metabolites, which are mainly metabolized into UA in the gut and the liver. Some foods increase UA levels more than others, such as meat, fructose, and alcohol [92,93,94]. Other foods can lower UA levels, such as milk, although the mechanisms are not fully understood. Perhaps, the link between milk consumption and PD risk may stand in its UA lowering properties. In fact, milk has an acute UA-lowering effect [95]: drinking cow’s milk lowers UA levels within hours, whereas drinking soy milk increases UA levels. The consumption of soy products results in different UA concentrations, with the highest levels of UA after soy milk intake, followed by soybean and soy powder intake [96].

High levels of UA, especially above 9 mg/dL, have been associated with a 22% increased risk of gout over five years, but when UA levels are below 7 mg/dL, the risk drops to 0.1% [97]. Besides gout, high blood UA levels have been associated with an increased risk of other conditions, such as hypertension, cardiovascular, and kidney diseases. Conversely, low UA levels have also been associated with an increased risk of Alzheimer’s Disease (AD), multiple sclerosis (MS), cancer, and stroke [98]. Although there is no specific UA target recommended for PD prevention, evidence suggests that maintaining UA blood concentrations within the upper physiological range (between 6 and 7 mg/dL) may exert neuroprotective effects on the central nervous system (CNS) while minimizing the risk of developing gout [60].

Dairy consumption may explain the differences between meat eaters, fish eaters, lacto-ovo-vegetarians and vegans. It is known that dairy products lower UA levels, probably because of their low purine content and the higher elimination of UA due to their protein content [95]. Results from the EPIC-Oxford study showed that vegans exhibited the highest UA serum levels compared to meat eaters, fish eaters, and lacto-ovo-vegetarians (mean UA levels in men: vegans 6.12 mg/dL; lacto-ovo-vegetarians 5.4 mg/dL; fish eaters 5.5 mg/dL; meat eaters 5.7 mg/dL; mean UA levels in women: vegans 4.3 mg/dL; lacto-ovo-vegetarians 4.1 mg/dL; fish eaters 4 mg/dL; meat eaters 4.2 mg/dL). This result was particularly evident among men. In contrast, lacto-ovo-vegetarians and fish eaters (excluding meat) had the lowest serum uric acid concentrations [99]. Vegans exhibited higher UA levels, likely due to their avoidance of dairy products. The lower content of calcium in a vegan diet might also contribute to explaining lower UA concentrations in some contexts. In line with the findings from the EPIC-Oxford study and various cross-sectional analyses, dietary calcium intake is inversely associated with UA levels [99,100]. Calcium supplementation was not associated with a reduction in UA concentration in a randomized controlled trial, but in that study, the mean calcium intake was higher compared to the average intake of calcium in vegans [101]. It is possible that calcium supplementation in people with lower calcium intakes, like vegans, would result in a reduction in UA concentration [99]. It is moreover possible that the consumption of soy products in vegans might explain higher UA levels, because plant purines might have a different effect on UA concentrations.

In summary, adherence to a vegan dietary pattern may maintain serum UA concentration within an optimal range, high enough to exert a neuroprotective effect, yet sufficiently controlled to mitigate the risk of metabolic comorbidities such as gout and cardiovascular disorders. Adherents to a plant-based dietary pattern are likely to maintain optimal UA concentrations, which may contribute to enhanced longevity and a reduced risk of age-related neurodegeneration. Further studies are needed to confirm these results and assess differences between men and women.

## 9. Flavonoids

Flavonoid compounds found in various plant-derived foods exhibit significant antioxidant and neuroprotective activities, contributing to the mitigation of neurodegenerative processes. Since α-synuclein misfolding and aggregation are key pathogenic processes in PD, different flavonoid compounds have been tested to identify which ones could inhibit α-synuclein fibril folding and aggregation. It was found that a variety of flavonoids (including flavones, flavonols, flavanones, isoflavones, dihydroflavonols, catechins, and anthraquinones) can inhibit α-synuclein fibrillation, and some were also able to disaggregate preformed fibrils [102]. In particular, flavonoids form non-covalent interactions with α-synuclein, restricting its conformational transition to β-sheet-rich fibrillar structures and stabilizing monomeric or oligomeric forms [103]. Some flavonoids, such as baicalein, also form covalent interactions with α-synuclein, further preventing fibril formation and promoting disaggregation [104]. α-synuclein plays a role in neurodegeneration through different mechanisms, including mitochondrial dysfunction; the possibility of preventing its aggregation and oligomerization is a promising strategy against neurodegeneration. Flavonoids are plant-derived phenolic compounds found in a variety of plant foods and beverages, including vegetables, fruits, nuts, grains, and black and green tea. Over 8000 flavonoid compounds have been identified to date, categorized into six major subgroups: flavones, flavanones, flavanols, flavonols, isoflavones, and anthocyanins. Flavonoids are typically consumed as glycosides, which are broken down by intestinal enzymes and the gut microbiota, releasing aglycones that are absorbed by intestinal cells. Aglycones are further metabolized in the liver and intestine, and their metabolites are quite rapidly excreted. Flavonoids have low bioavailability because they are poorly absorbed and rapidly metabolized and excreted, but they can bind α-synuclein soluble monomers and oligomers within the intestinal lumen and stabilize them. Among neuroprotective flavonoids, epigallocatechin-3-gallate has been shown to protect against dopaminergic degeneration [105]. The most studied flavonoid in PD is baicalein, isolated from the roots of *Scutellaria baicalensis* and *Scutellaria lateriflora*, which has antioxidant, anti-inflammatory, and neuroprotective properties against PD. Baicalein can directly increase dopamine levels, and reduce the amount of misfolded α-synuclein [106,107]. The synergistic interaction between baicalein and levodopa can reduce the adverse effects associated with high levodopa doses [108].

Naringin-7-O-glycoside, a major flavanone abundant in *Citrus* species, exhibits strong neuroprotective effects in PD by modulating oxidative and inflammatory pathways. Beyond its antioxidant capacity, naringin may further support neurorestoration by enhancing dopamine synthesis and preserving dopaminergic integrity [109]. Another neuroprotective flavonoid is kaempferol, found mainly in citrus fruits, strawberries, apples, beans, broccoli, onions, and tea. It exerts its beneficial effect by modulating pro-inflammatory signaling pathways, reducing reactive oxygen species (ROS), and modulating brain-derived neurotrophic factor (BDNF) [110]. A study by Han et al. found that kaempferol can prevent dopaminergic neuron degeneration by inhibiting lipid peroxidation-induced mitochondrial damage, offering a potentially novel therapeutic approach for PD [111]. In addition, it has been shown that kaempferol and its metabolites can directly reduce α-synuclein aggregation [112]. Anthocyanins represent the most significant flavonoid subclass associated with a reduced risk of PD. These phytochemicals are found in high concentrations in various berries, including blackcurrant (*Ribes nigrum*), cherry (*Prunus avium*), blueberry (*Vaccinium* spp.), mulberry (*Morus* spp.), and black elderberry (*Sambucus nigra*) [113]. Anthocyanins include: cyanidin, proanthocyanidin, delphinidin, malvidin, pelargonidin, and petunidin. Extracts rich in anthocyanins can elicit greater neuroprotective effects than other polyphenols. They can ameliorate mitochondrial function, increase antioxidant effects by scavenging ROS, reduce membrane damage, and rescue dopaminergic neurons [113]. Notably, some cohort data suggest a more pronounced protective effect in men; however, it is unclear if this indicates sexual dimorphism in neuroprotection, as stratified analyses of specific flavonoid subclasses have yielded inconsistent results regarding sex-specific differences [114].

Although epidemiological studies have shown an inverse relationship between flavonoid intake and the incidence of chronic diseases, including neurodegenerative disorders, little is known about the exact mechanisms through which they exert their benefits. The main hypothesis is that they counteract oxidative stress by scavenging radicals and through metal chelation, although their direct contribution may be relatively low because most circulating flavonoids are metabolites with reduced antioxidant properties [115]. An alternative hypothesis proposes that these phytochemicals can modulate specific intracellular signaling cascades, a hypothesis consistent with the documented bioactivity of various flavonoid-derived metabolites in neuroprotection [116]. Some flavonoids, like curcumin and kaempferol, can exert their beneficial role through epigenetic effects such as DNA methylation, histone modifications, and increasing non-coding RNAs [117]. Additionally, a significant factor is the capacity of flavonoids to promote gut microbiota homeostasis. Intestinal microbiota metabolizes flavonoids, increasing their bioavailability; in turn, phytochemicals modulate the taxonomic composition and functional structure of the gut microbiota [118]. Flavonoids are thought to have properties similar to those of prebiotics, as they feed gut commensal bacteria [118]. Metabolites of prebiotic flavonoids, such as short-chain fatty acids (SCFAs) (propionate, acetate, and butyrate), can reduce gut inflammation by enhancing epithelial barrier integrity [119]. Flavonoids are typically transformed into bioactive metabolites by the gut microbiota and, for this reason, their bioavailability is determined by their catabolism. After portal translocation, xenobiotic biotransformation occurs in the liver before they can reach the bloodstream as phenolic metabolites [119]. Anthocyanins have shown neuroprotective properties thanks to gut microbiota modifications, with consequent modified tryptophan metabolism and the generation of kynurenic acid, a neuroprotective compound [120]. Also, catechins are able to modify the gut microbiota, reducing the number of pathogenic bacteria such as *Helicobacter pylori*, *Escherichia coli*, and *Listeria monocytogenes* and increasing the number of beneficial strains such as *Actinobacteria* and *Verrucomicrobia* [121].

A plant-based diet rich in flavonoids can be beneficial in PD by increasing dopamine levels and reducing ROS and α-synuclein aggregation, with consequent motor improvement. Further studies are needed to clarify the bioavailability, safety and effective doses of flavonoids [122].

## 10. Coffee

Coffee is the most commonly consumed beverage worldwide, with well-known psychostimulant effects thanks to the presence of caffeine (1,3,7-trimethylxanthine), a natural alkaloid with bioactive properties. The majority of coffee beverages derive from *Coffea arabica* (Arabica) and *Coffea canephora* (Robusta) species, which differ in caffeine content, with Robusta beans containing the highest levels. Coffee’s beneficial effects can also derive from other bioactive compounds with antioxidant properties such as phenolic compounds (e.g., chlorogenic acids), diterpenes (cafestol and kahweol), nicotinic acid (Vitamin B3), and other secondary metabolites [123]. Drinking coffee regularly can reduce the risk of heart, vascular and liver diseases, and it can also improve asthma symptoms. Moreover, regular coffee consumption appears to be associated with a reduced severity of some neurodegenerative disorders [124]. Different prospective studies have demonstrated that drinking coffee is associated with a 50% reduction in the risk of developing PD [125]. Similar results have also been confirmed by the EPIC4PD study [126]. Caffeine intake may also slow down disease progression, especially when combined with physical activity [127,128]. PD patients who consume coffee or caffeinated tea have a 50% reduced risk of all-cause mortality [125]. A meta-analysis of 13 studies involving 901,764 participants found a non-linear association between coffee consumption and PD risk, with maximum protective effects observed at about three cups per day [129].

Caffeine is an adenosine A2A receptor antagonist; these receptors are densely located on dopaminergic neurons. The activation of these receptors induces neural excitotoxicity with consequent neuronal death. By antagonizing this receptor, caffeine could play a neuroprotective role, reducing hypercalcemia-related damage and inflammation [130]. Some alkaloids, like caffeine, can inhibit α-synuclein aggregation, preventing dopaminergic neuron degeneration [131]. Other compounds, like chlorogenic acid, can reduce dopaminergic neuron loss [64]. Diterpenes, like kahweol and cafestol, can promote neuroprotection through their antioxidant activity [132]. Moreover, coffee consumption can regulate the gut microbiota by promoting the growth of anti-inflammatory *Bifidobacterium* and decreasing negative strains like *Clostridium* spp. and *Escherichia coli* [133]. Coffee can be beneficial for cognition as well: neuropsychological research has shown that coffee drinking improves cognitive performance in elderly people with metabolic syndrome [134]. Moreover, coffee consumption can also positively affect mood, as shown in a cross-sectional study conducted on 196 PD patients [135]. Regarding the beneficial effects of coffee consumption on motor PD symptoms, a study conducted on 284 patients with de novo PD found a negative correlation with tremor severity, but only in male patients [136]. A randomized controlled trial conducted for 6 weeks on 61 PD patients by Postuma et al. found that regular caffeine consumption (200 mg twice a day, equivalent to four cups of coffee/day or six cups of green tea/day) was associated with improved motor skills, as assessed by the Unified Parkinson’s Disease Rating Scale III (UPDRS-III) [137]. In contrast, a more recent multi-center randomized controlled trial conducted over 6–18 months on 121 PD patients, found that regular caffeine consumption (200 mg twice a day) was not associated with significant motor improvement [138].

In summary, coffee consumption seems to be protective against the development of PD and has many health benefits, including the promotion of a more beneficial gut microbiota. Coffee may also be beneficial for cognitive function and mood; however, no sustained benefit has been observed regarding motor symptoms in PD patients, likely due to its short-term effects, which wear off too rapidly. For this reason, regular coffee consumption cannot be recommended as part of symptomatic therapy in PD.

## 11. Nicotine and Other Components of Edible *Solanaceae* and Tobacco

Previous studies have shown that smoking can protect against PD. A meta-analysis found that ever-smokers had a 41% lower risk of developing PD compared to never smokers, and that current smokers had a 58% risk reduction. A dose-dependent relationship was also found, with higher smoking exposure linked to a greater risk reduction [139]. A case–control study found that passive smoking was also associated with a 66% reduction in PD risk, suggesting that smoke exposure itself, rather than the act of smoking, is protective [140]. In summary, multiple studies have demonstrated a 30–60% reduction in PD risk among smokers compared to non-smokers. This appears to be a dose-dependent association, where higher cumulative smoking exposure was associated with a further decrease in risk [141]. The exact mechanisms through which smoking could be protective against PD are not completely clarified, as tobacco is heterogeneous in its chemical composition [142]. Tobacco can inhibit monoamine oxidase (MAO), which causes an enhancement of dopaminergic neurotransmission [143]. The inhibition of MAO-B also reduces ROS and consequently displays an antioxidant protective effect [144]. Other studies have focused on the role played by nicotine in eliciting neuroprotective effects on dopaminergic neurons [145]. Nicotine and hydroquinone, another compound contained in smoke, can inhibit α-synuclein aggregation by stabilizing the soluble oligomeric form of the protein [146].

It has been well established that smoking is harmful, since it is associated with an increased risk of many chronic diseases, including cardiovascular diseases and cancer; this negative effect far outweighs its neuroprotective benefits against PD. For this reason, tobacco has been studied to identify potential neuroprotective components that can also be found in food. In particular, nicotine has also been found in the *Solanaceae* family, including edible fruits and tubers such as peppers, tomatoes, potatoes, and eggplants [147], athough the amount of nicotine absorbed from these foods is lower than that obtained through active smoking. Nicotine has also been detected in the toenails of non-smokers, albeit to a lesser extent than in smokers [148]. This suggests that nicotine is absorbed through the daily diet, and indeed, an inverse association has been observed between PD and the consumption of *Solanaceae* vegetables [147]. Fresh edible *Solanaceae* contain measurable nicotine levels (2–7 mcg/kg), although these are significantly lower than the amount found in a single cigarette (~1 mg). Nicotine has also been found in black and green teas with a variable content, which in some cases is higher than that detected in edible *Solanaceae*. The average daily dietary intake of nicotine was estimated at 1.4 mcg/day, reaching 2.25 mcg/day at the 95th percentile [149]. Nicotine receptors are highly saturable, and a small amount of nicotine is sufficient to reach its effect [150]. Peppers (*Capsicum annuum*) have the highest amount of nicotine among edible *Solanaceae* (102.1 mcg/kg). Eating them two to four times a week could confer an over 30% increase in protection against PD. The effects of eating nicotine-containing foods were more evident in non-smokers than in smokers, as the nicotine from smoke would mask the dietary benefits [147]. The contribution of dietary nicotine intake is still significant when compared to passive and active smoking, and this could explain why diets rich in edible *Solanaceae*, like the Mediterranean diet, could be protective against PD.

Besides nicotine, other *Solanaceae* components from pepper may be preventive against PD: anatabine is another alkaloid exerting anti-inflammatory properties and a potentially neuroprotective action. When compared to nicotine, anatabine was found to have a longer half-life, lower toxicity, and a higher anti-inflammatory effect [151]. Capsinoids found in peppers (including capsaicinoids found in spicy peppers) could increase midbrain dopaminergic neuron survival [152]. Other components found in *Solanaceae* fruits, such as lycopene, vitamin C, and vitamin A, are unlikely to explain the protective effects of these foods against PD. This highlights the importance of phytochemical components like anatabine and capsaicinoids in PD prevention, which need to be addressed in further studies.

All these findings raised the question of whether nightshade vegetables could help in treating PD symptoms. Results from clinical trials and case studies have demonstrated that nicotine was capable of improving PD symptoms (observed in 5 out of 10 studies). The reason for these mixed results was due to variations in the mode of administration (patch, gum, intravenous), dosing, treatment timing and duration. For this reason, there are doubts regarding a direct beneficial effect of nicotine on motor PD symptoms [153].

Further studies are needed to strengthen causal inferences about the role of edible *Solanaceae* in PD prevention and to test the efficacy of these foods in PD treatment. However, considering that *Solanaceae* edible fruits and tubers are healthy and tasty, they offer more benefits than potential risks; thus, it is advisable to include them regularly in the diet.

## 12. Dietary Patterns and Parkinson’s Disease Risk

Observational studies have associated some dietary patterns and specific food groups with various aspects of PD, including its risk, age of onset, progression, symptom severity, and mortality rates, although randomized controlled trials (RCTs) on nutritional intervention in PD are scarce. Nutritional interventions can potentially interfere with multiple disease mechanisms and they might modify the disease course. Moreover, a proactive management of dietary habits can empower PD patients to take control of an aspect of their care, improving their quality of life and influencing symptoms management.

Listed below is the current evidence on dietary pattern in PD:

### 12.1. Mediterranean Diets

Mediterranean diets (MeDi) are predominantly composed of plant foods. They include a variety of vegetables, fruits, whole grains, legumes, unsaturated fatty acids (especially from olive oil), nuts, seeds, and fish or seafood. They also allow for a moderate consumption of poultry and wine while limiting red and processed meats, refined grains, sugar, and saturated fats. This dietary pattern is associated with many health benefits, as it improves metabolic and cardiovascular health and reduces inflammation [154]. A reduced PD risk, a later age of onset, and fewer PD prodromal symptoms have been reported in patients following a MeDi, suggesting that this kind of diet might be protective [155]. Its protective effects stem from the content of foods with anti-inflammatory and antioxidant properties, such as phytochemicals (including polyphenols), omega-3 fatty acids, and fiber, which are beneficial to the gut microbiome [155]. Despite this evidence, randomized controlled trials (RCTs) are limited. A similar dietary pattern is the Japanese one, which is mainly plant-based, and contains high amounts of fruits, vegetables, and fish. In a case–control study, this pattern was found to be protective against PD [156].

### 12.2. Ketogenic Diets

A ketogenic diet (KD) is a high-fat, low-carbohydrate diet that generates ketone bodies such as beta-hydroxybutyrate, which can be used by the brain as an energy source. This mechanism may bypass a bioenergetic deficit in PD, where damaged neurons cannot efficiently use glucose as a source of energy [157]. It is hypotesized that ketone bodies ameliorate mitochondrial function [158]. Research on the effects of ketogenic therapy in PD is still in its early stages. RCTs on ketogenic diets in PD patients are very scarce and heterogeneous, with no conclusive results [159]. Some studies show little benefit on some motor and non-motor PD symptoms [160,161].

### 12.3. MIND (Mediterranean-DASH Intervention for Neurodegenerative Delay) Diet

The MIND was designed to protect against neurodegeneration and integrates elements of the MeDi and the Dietary Approaches to Stop Hypertension (DASH). This dietary pattern is highly plant-based, emphasizing the consumption of green leafy vegetables and berries, and has been associated with a reduced risk of all-cause mortality [162], and cognitive decline [163]. A reduced risk of PD has been reported with this diet [164], although this association was more substantial in the female subgroup. One study found the MIND diet to be more protective against PD than the MeDi [165]. However, the impact of the MIND diet on PD symptoms, progression, and PD-associated cognitive decline has yet to be assessed through interventional studies.

### 12.4. Vegetarian (Lacto-Ovo-Vegetarian and Vegan) Diets

Vegetarian diets have been associated with a reduced risk of all-cause mortality, including PD, especially in younger populations. Data from the Adventist Health Study-2 cohort showed that vegetarian diets are associated with lower all-cause mortality, especially for cardiovascular disease and cancer [166]. Previous epidemiological studies have also shown that predominantly vegetarian cultures have lower rates of PD [167]. A UK Biobank analysis identified an association between a healthy plant-based diet and a reduced risk of PD, whereas an unhealthy plant-based diet was linked to an increased risk of PD [168]. A prospective cohort study performed on the UK biobank showed a reduced risk of AD and PD with increased physical activity, especially when combined with a plant-based diet [169]. A small trial on PD patients following a vegetarian diet showed improvement in UPDRS Part III scores [170]. During the 20th century, populations in East Asia and sub-Saharan Africa consuming an almost vegan diet were found to be at decreased risk of PD compared with US and European populations. It has been proposed that such diets may provide protection from PD by boosting mitochondrial function through an enhanced expression of Parkin and PINK1 in the SN [171]. A prospective study that analyzed the association between different dietary patterns and the risk of developing PD found that subjects with a high intake of fruits, vegetables, legumes, whole grains and nuts, paired with a low intake of saturated fat, may be protected against PD [172]. A small pilot study has also demonstrated that plant-based diets are beneficial for PD, especially in the management of the disease. In fact, in this study, PD patients on a plant-based diet demonstrated a significant improvement in motor performance, as evidenced by the UPDRS III total score and the modified H&Y Staging Scale [173].

Evidence suggests that plant-based patterns seem to be protective against PD and can also reduce disease severity. In addition, they carry many other health benefits, such as a reduction in the risk of other chronic disorders, particularly cardiovascular and cerebrovascular ones. Larger high-quality randomized controlled trials are warranted to define which dietary pattern is most beneficial in preventing PD.

**Figure 2:**
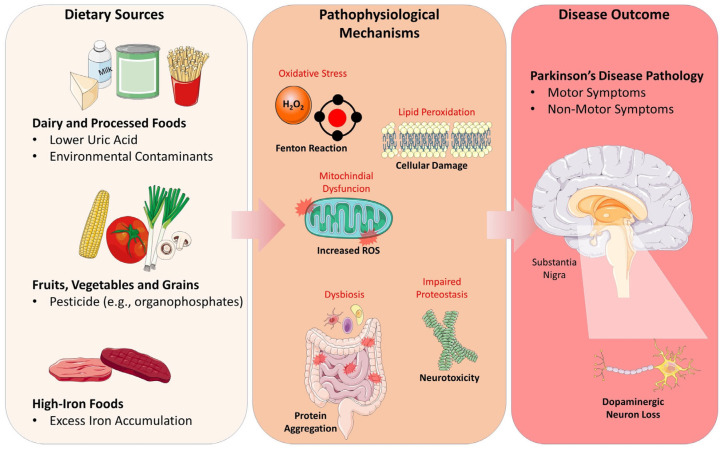
Proposed dietary mechanisms contributing to Parkinson’s Disease Pathology.

Figure 2 illustrates the proposed dietary and pathophysiological mechanisms underlying Parkinson’s Disease pathology.

## 13. Foods Containing Dopamine and Levodopa

Some plant foods contain dopamine. For example, Cavendish bananas contain between 2.5 and 10 mg/100 g of dopamine in the pulp and 80–560 mg/100 g in the peel [174]. Avocado contains between 0.4 and 0.5 mg/100 g of dopamine, whereas other plant foods, such as oranges, tomatoes, aubergines, spinach, peas and wild apples, contain only trace amounts [175]. However, dietary dopamine cannot cross the BBB and, consequently, cannot exert its effect within the brain. Only levodopa (L-DOPA), a dopamine precursor, can cross the BBB to be converted into dopamine in the brain by the enzyme DOPA-decarboxylase.

Ancient Indian texts, dating back thousands of years, documented that individuals suffering from tremors, rigidity, and gait disorders were treated with plant food belonging to the *Fabaceae* family [176]. Faba beans (*Vicia faba*, VF), also known as Broad Beans, are seeds of a leguminous plant. Guggenheim in 1913 identified the presence of levodopa in the seeds, pods, and beans of VF [176]. VF can contain a variable amount of levodopa, depending on different factors, such as species, climate zone, soil conditions, and precipitation. The amount of levodopa in VF is enough to be pharmacologically active in PD [177]. In 1993, a small study conducted on five controls and six PD patients demonstrated that VF ingestion produced a significant increase in levodopa plasma levels, which correlated to improved motor function [176]. Participants ingested 250 g of cooked broad beans. After 12 h off-treatment, PD patients exhibited significant motor improvement over the following four hours; however, half of the participants developed marked dyskinesias. PD motor improvement was similar to a treatment with 125 mg of levodopa combined with 12.5 mg of carbidopa [178]. Fresh green VF contain more levodopa than dried VF, and roasting or boiling processing reduces the levodopa content [179,180]. Half a cup of cooked VF (100 g) contains approximately 250 mg of levodopa, and sprouted VF beans contain the highest levodopa concentrations [181]. VF does not contain only levodopa but also carbidopa, and this contributes to the motor improvement [182].

The primary challenge is that treating PD patients requires large quantities of VF to be consumed, which may cause side effects, including flatulence and abdominal bloating. VF is also rich in tyramine, and for this reason, it should be consumed carefully with concomitant MAO inhibitor (I-MAO) therapy, often taken by PD patients. In fact, this interaction could lead to a dangerous elevation in blood pressure [183]. Nevertheless, since VF contains appreciable amounts of levodopa, a sudden interruption of its consumption might trigger a neuroleptic malignant-like syndrome, as has been reported in the literature, indicating that alternative therapies share similar risks to conventional ones, so caution is always needed [181]. Additionally, PD patients affected also by favism cannot consume VF because of the lack of glucose-6-phosphate dehydrogenase (G6PD).

Other beans, such as common beans, green beans and soy beans, contain levodopa at a lower amount. *Phaseolus vulgaris*, a common bean, contains 0.25 g/100 g of levodopa, although studies on its use in PD are lacking [184]. Soybeans contain 0.02 g/100 g of levodopa, but they contain genistein, an isoflavone, which inhibits DOPA-decarboxylase, increasing the bioavailability of levodopa [185]. In PD patients, the consumption of 11 g of roasted soybeans as an adjunct to levodopa/carbidopa treatment led to significant motor improvement compared to synthetic levodopa alone, while also reducing dyskinesias [186]. Cocoa beans contain phenylethylamine, a dopamine precursor, although no motor improvement has been reported in PD patients treated with dark chocolate [187]. Another bean that has been recognized to be beneficial in PD is Velvet beans (*Mucuna pruriens*, MP), a tropical legume, belonging to the family of *Fabaceae*, native to Africa and tropical Asia but cultivated nowadays in different countries. They are used in Ayurveda medicine as anti-inflammatory agents and are also used in Asian traditional medicine, especially in India, to treat different diseases, including neurodegenerative diseases like PD. MP contains about 10 times more levodopa compared with VF, which represents the highest amount of levodopa among all plant foods [184]: for this reason, it can be eaten as food in place of a supplement. All parts of MP have medicinal properties, particularly neuroprotective effect. Specifically, this legume has potential therapeutic effects in PD as its seeds contain high doses of levodopa (4–7% of its weight), similar to that contained in synthetic levodopa drugs [188]. MP levodopa seems to be more effective than standard levodopa, probably due to an unknown dopamine decarboxylase inhibitor contained in the bean [189]. However, the bioavailability of levodopa from velvet beans may vary depending on different factors, including preparation methods. The effectiveness of MP in improving PD symptoms and its safety has been demonstrated in several open-label randomized clinical trials [190,191]. A double-blind clinical and pharmacological study tested the efficacy of MP compared to standard levodopa/carbidopa therapy, and it was found that 30 g of MP can lead to a more rapid effect and a shorter latency to levodopa peak, without concomitant dyskinesias. The fact that MP exerts the same effect as levodopa with fewer side effects, such as dyskinesias, highlighted the possible advantages of such therapy in place of standard levodopa, especially in the long-term management of PD [192]. In another small double-blind, randomized, controlled, crossover study, performed on 18 PD patients, the administration of 12.5 mg/kg of MP showed a similar effect to the one induced by dispersible levodopa/benserazide (3.5 mg/kg), but with fewer dyskinesias and adverse events. The administration of a higher dose of MP (17.5 mg/kg) resulted in better motor improvement and longer ‘ON’ duration, while simultaneously reducing the incidence of dyskinesias and adverse events. The conclusion of this study was that single-dose MP intake has an analogous efficacy and safety compared to dispersible levodopa/benserazide. Clinical effects of high-dose MP are similar to those obtained through levodopa, but with higher tolerability [193]. A 16-week open-labeled phase 2 pilot trial was performed on 14 Bolivian PD patients in an advanced stage of the disease. These patients were treated with a daily dose of MP. Fifty percent of these patients withdrew from the trial prematurely due to gastrointestinal side effects and motor worsening; however, the remaining 50% showed a motor response comparable to that obtained using standard levodopa/carbidopa. The overall benefit of MP was limited by its scarce tolerability [194]. These small studies highlight that further investigation is needed on larger cohorts of patients to assess the safety and efficacy of MP, especially in the long term.

PD therapies are still unavailable and unaffordable in most Low-Income Countries, especially synthetic levodopa, and MP may be considered in these countries as an alternative to standard drug therapy used for the treatment of PD [195]. Roots and seeds of MP are rich in terpenes, sterols (β-sitosterol, ursolic acid, etc.), NADH, and Coenzyme Q10 [196,197]. These compounds exert a potent antioxidant and anti-inflammatory effect [198,199]. Due to the natural variability in its levodopa content, standardizing the dosage of MP remains an important challenge. MP can also interact with other medications [194] and it can be harmful when consumed in large amounts, so medical surveillance is mandatory. Common side effects reported in patients are gastrointestinal, like nausea, vomiting, and loss of appetite, but also neuropsychiatric, like paranoid delusions, hallucinations, and delirium. Currently, there is insufficient data regarding the long-term effects of MP and consequently, a cautious approach in its clinical application is warranted [184]. Therefore, although promising, MP cannot actually be considered a standardized therapy for PD.

In summary, although velvet beans are promising alternative choices for PD treatment, their use has not yet been standardized in the clinical setting, and other studies are needed to define their safety, efficacy, and long-term effects. Levodopa/carbidopa and levodopa/benserazide remain the gold-standard therapy for PD, although regular consumption of a variety of beans may provide further benefits.

## 14. An Ideal Nutritional Approach to PD Patients

It is essential for PD patients to follow a well-planned diet, as food can affect both the pharmacokinetics and pharmacodynamics of levodopa: the most effective symptomatic treatment for the disease. Specifically, meals can slow gastric emptying, thereby delaying levodopa absorption in the small intestine. On the other hand, large neutral amino acids (LNAAs) compete with its saturable transport system in the intestine. The LNAA L-leucine decreases the intestinal absorption of levodopa by 50% [200]. Levodopa also competes with a highly saturable LNAA transporter located at the BBB, and it is the competition at this site that mainly contributes to the variability in levodopa pharmacokinetics [201]. The main determinant of LNAA plasma levels is protein loading, and for this reason, the diet should limit protein intake to the recommendation for the general population. Ideally, the diet for PD patients should be normoproteic (protein around 10–15% of total calories with about 0.8–1.0 g/kg/day), avoiding further limiting of the protein intake, because protein deficiency can lead to malnutrition and sarcopenia [173]. In order to maximize levodopa efficacy, a redistribution of protein in meals, concentrating most of it at dinner, might be a reasonable approach [202]. In addition, the diet should be rich in insoluble fiber to increase levodopa bioavailability with a consequent improvement in motor function [203]. Proteins from plant-based foods, such as legumes and vegetables, should be preferred as they also contain fiber, which helps regulate gastrointestinal transit. Conversely, a high intake of animal proteins, such as in meat-based diets, can interfere with levodopa absorption and bioavailability, often correlating with increased motor fluctuations throughout the day [204]. A pilot study by Baroni et al., performed on 25 PD patients for four weeks, reported that a vegan, plant-based diet incorporating a protein-redistribution strategy significantly improved both the stage (Hoehn and Yahr Staging Scale, 1.96 vs. 3.15, *p* = 0.005) and the UPDRS total score (47.67 vs. 74.46, *p* = 0.008) and UPDRS Part III motor performances (25.42 vs. 46.46, *p* = 0.001). This dietary intervention effectively minimized the competitive interference between dietary amino acids and levodopa absorption during active daytime hours [173]. Indeed, a plant-based diet, especially in its vegan variant, can increase levodopa bioavailability also thanks to a higher fiber content when compared to omnivorous diets. Beyond dietary composition, levodopa should be taken on an empty stomach, ideally 60 min before a meal or 2 h after a meal. It is important that PD patients receive adequate nutritional counseling in order to plan the most suitable diet for the disease but also for their lifestyle and personal taste. Challenges to dietary adherence include chemosensory alterations, such as hyposmia, which can reduce appetite, gastrointestinal dysmotility and nausea that can also reduce food intake. In addition to that, advanced PD patients often have dysphagia, which is an important contributor to malnutrition. Dietary choices are also influenced by cultural food preferences, comorbidities and social factors. A personalized diet in PD should account for all these factors and routine nutritional assessment is mandatory to prevent malnutrition and optimize levodopa pharmacokinetics. The nutritional approach should always be personalized to increase patients’ compliance and their overall quality of life.

The core neurodegenerative processes of Parkinson’s Disease (PD) are summarized in Table 1, while the specific nutritional risk factors and protective dietary elements, alongside their respective pathophysiological mechanisms, are detailed in Table 2 and Table 3.

## 15. Limitations

This review has included *in vitro* studies and human trials, while excluding animal studies. The limitation of *in vitro* studies is that they lack the complex physiological context of the human body, especially gut–central nervous system interaction. Furthermore, nutrient metabolism and bioavailability in vivo cannot be replicated. Moreover, *in vitro* experiments cannot highlight genetic variability and individual responses to dietary interventions. Trials on humans are limited by small sample sizes, short intervention durations, and heterogeneous dietary interventions and outcome measures. This heterogeneity may not lead to strong meta-analysis, making it difficult to draw firm conclusions about the efficacy of specific dietary patterns or nutrients in modifying disease progression or symptoms. Larger high-quality randomized controlled trials are necessary to define which food and dietary pattern are more protective against PD, including plant-based diets.

## 16. Conclusions

PD ranks among the most prevalent neurodegenerative disorders globally, and nutrition may represent a pivotal modifiable risk factor. The current medical literature consistently demonstrates strong evidence for dairy intake as a risk factor for PD, with multiple large prospective cohort studies and meta-analyses showing a dose-dependent increase in risk. Strong evidence also exists for low UA levels and pesticide intake as risk factors for PD, whereas for other foods, such as meat and saturated fat, the evidence is less consistent and generally weaker, with some studies suggesting a possible increased risk for PD. Moderate-to-strong evidence also exists for coffee consumption as a protective dietary factor against PD. The evidence regarding plant-based foods and in general healthy dietary patterns (Mediterranean diet, MIND) as protective against PD is moderate. In conclusion, a diet excluding animals and animal-derived products, specifically dairy products, may lower exposure to several dietary risk factors linked to the pathogenesis of PD. Moreover, plant foods contain many neuroprotective compounds, such as flavonoids and fiber, and they are more sustainable. Besides neuroprotective effects, a high intake of plant foods and a low intake of animal foods can improve PD symptoms such as constipation and levodopa absorption. Adherence to a well-planned plant-based diet, particularly a vegan pattern, can ensure adequate protein intake without competing with levodopa absorption. Although further studies are required to clarify which specific phytonutrients exert the most significant neuroprotective effects, a balanced plant-based diet can help prevent various chronic diseases. Furthermore, PD patients may benefit from the consumption of plant-based foods, which exert beneficial effects on both symptom management and disease progression.

## Figures and Tables

**Table 1:** Parkinson’s Disease Neurodegenerative mechanisms.

Mechanisms	Description	Consequences
Dopaminergic neuron loss	Degeneration of dopaminergic neurons located in the SNpc of the mesencephalus.	Striatal dopamine loss and PD motor and non-motors symptoms development [8].
α-synuclein aggregation	Pathologic misfolded α-synuclein aggregation form Lewy bodies [205].	Synaptic dysfunction and neuronal death [206].
Oxidative stress	ROS formation and oxidation of macromolecules including lipids, proteins and nucleic acids [207].	Mitocondrial dysfunction, neuroinflammation and neuronal damage [208].
Mitochondrial dysfunction	Impaired endo-lysosomal-proteasome system functioning [209].	Decline mitochondrial bioenergetics and the subsequent triggering of stress-mediated apoptotic cascade [210].
Endolysosomal impairment	Caused by oxidative stress, GBA1 mutations and α-synuclein aggregation [211].	Disruption of proteostasis, autophagy, and mitophagy, leading to the accumulation of misfolded proteins such as α-synuclein and damaged mitochondria with consequent neurodegeneration [212].
Synaptic dysfunction	As a consequence of the aforementioned mechanisms but especially α-synuclein deposition [213].	Axonal damage, connectome dysfunction and neurodegeneration [213].

**Table 2:** Nutritional risk factors and related mechanisms linked to Parkinson’s Disease.

Nutritional Risk Factors	Mechanisms
Meat and its derivatives	Several dietary factors linked to animal-based products have been implicated in PD, including saturated fatty acids (SFAs) [18] and heterocyclic amines (HCAs), which demonstrate specific neurotoxicity toward dopaminergic (DA) neurons [22]. Additionally, these dietary sources may facilitate the introduction of exogenous α-synuclein [23] and promote gut microbiota dysbiosis [26], thereby fostering a pro-inflammatory environment conducive to neurodegeneration.
Seafoods	Chronic exposure to elevated levels of heavy metals may significantly contribute to PD pathogenesis through several mechanisms. Specifically, these contaminants exhibit high affinity for dopamine D2 receptors [39], and promote the misfolding and presynaptic aggregation of α-synuclein [40]. Furthermore, heavy metal-induced disruption of blood–brain barrier (BBB) integrity [41] exacerbate neuronal vulnerability by allowing systemic toxins penetrating the brain parenchyma.
Dairy products and low UA levels	The association between dairy intake and PD may be driven by a synergistic toxicological profile. This includes exposure to bioaccumulated pesticides and D-galactose induced mitochondrial dysfunction [58]. Moreover, dairy protein intake can lead to a significant reduction in serum uric acid (UA) concentrations [59], reducing the amount of circulating antioxidants that can counteract dopaminergic loss.
Dietary fat intake	Dietary saturated fatty acids (SFAs) can act as potent triggers for low-grade chronic inflammation [19,61]. This proinflammatory environment fuels oxidative stress [63], further contributing to DA neuron degeneration.
Pesticides	Some pesticides can act like neurotoxicants: paraquat, rotenone, and several organochlorines have been identified as significant environmental risk factors for PD [71,72]. Their mechanisms of action include mitochondrial dysfunction, inflammation, DNA methylation, altered calcium transient [75] and intestinal dysbiosis [76], with all mechanisms further contributing to neurodegeneration.

**Table 3:** Nutritional protective factors and related mechanisms linked to Parkinson’s Disease.

Nutritional Protective Factors	Mechanisms
Flavonoids	Neuroprotective phytonutrients are capable of reducing α-synuclein aggregation [103] and mitigating oxidative stress. These compounds also promote a healthy gut microbiota composition [123]. Specifically, anthocyanins exhibit significant neuroprotective properties toward dopaminergic (DA) neurons by enhancing mitochondrial bioenergetics, scavenging reactive oxygen species (ROS), and preserving membrane integrity, which collectively contribute to the rescue of DA neurons in PD models [114].
Coffee	The neuroprotective role of caffeine is primarily attributed to its antagonism on adenosine A2A receptors, a mechanism that mitigates neuroinflammation and excitotoxicity in DA neurons [131]. Additionally, caffeine can contribute to proteostasis by inhibiting α-synuclein aggregation [132], and can enhance cellular defenses through its intrinsic antioxidant properties [133].
Edible *Solanaceae*	Nicotine has been found to suppress α-synuclein aggregation [147], while various other alkaloids identified in dietary sources demonstrated significant neuroprotective and anti-inflammatory properties [146,153].

## Data Availability

The original contributions presented in this study are included in the article. Further inquiries can be directed to the corresponding authors.

## Abbreviations

The following abbreviations are used in this manuscript:

PD	Parkinson’s Disease
LBD	Lewy Body Dementia
LNAAs	Large Neutral Amino Acids
VF	*Vicia faba*
MP	*Mucuna pruriens*
BBB	Blood–Brain Barrier
UA	Uric Acid
